# Early Vocabulary in Relation to Gender, Bilingualism, Type, and Duration of Childcare

**DOI:** 10.5709/acp-0192-6

**Published:** 2016-09-30

**Authors:** M. Stolarova, A. A. Brielmann, C. Wolf, T. Rinker, T Burke, H. Baayen

**Affiliations:** 1 Department of Children and Child Care, German Youth Institute, Munich, Germany; 2 Department of Psychology, New York University, New York City, NY, USA; 3 Department of Psychosomatic Medicine, Kantonssital St. Gallen, St. Gallen, Switzerland; 4 Department of Linguistics and Zukunftskolleg, University of Konstanz, Konstanz, Germany; 5 Department of Family Relations and Applied Nutrition, University of Guelph, Canada; 6 Department of General Linguistics, University of Tübingen, Tübingen, Germany

**Keywords:** vocabulary acquisition, language development, conceptual processing, early childhood education, ELAN, gender similarities, bilingual development

## Abstract

This study investigates the predictive value of child-related and environmental characteristics for early lexical development. The German productive vocabulary of 51 2-year-olds (27 girls), assessed via parental report, was analyzed taking children’s gender, the type of early care they experienced, and their mono- versus bilingual language composition into consideration. The children were from an educationally homogeneous group of families and state-regulated daycare facilities with high structural quality. All investigated subgroups exhibited German vocabulary size within the expected normative range. Gender differences in vocabulary composition, but not in size, were observed. There were no general differences in vocabulary size or composition between the 2 care groups. An interaction between the predictors gender and care arrangement showed that girls without regular daycare experience before the age of 2 years had a somewhat larger vocabulary than all other investigated subgroups of children. The vocabulary size of the 2-year-old children in daycare correlated positively with the duration of their daycare experience prior to testing. The small subgroup of bilingual children investigated exhibited slightly lower but still normative German expressive vocabulary size and a different vocabulary composition compared to the monolingual children. This study expands current knowledge about relevant predictors of early vocabulary. It shows that in the absence of educational disadvantages the duration of early daycare experience of high structural quality is positively associated with vocabulary size but also points to the fact that environmental characteristics, such as type of care, might affect boys’ and girls’ early vocabulary in different ways.

## Introduction

 Early vocabulary acquisition is influenced by complex interactions of biological, socio-economic, and learning factors (Gervain & Mehler, 2010 ; Stokes & Klee, 2009). They often affect both quality and quantity of the language input children receive (Bohman, Bedore, Peña, Mendez-Perez, & Gillam, 2009 ; Hammer et al., 2012 ; Harris, Golinkoff, & Hirsh-Pasek, 2010 ; Hart & Risley, 2003 ; Hoff, 2006 ; Rohacek, Adams, & Kisker, 2010). Vocabulary size is highly predictive for further language development (Fernald & Marchman, 2012 ; Lee, 2011 ; Marchman & Fernald, 2008), and it is also considered an important predictor for later educational success (Walker, Greenwood, Hart, & Carta, 1994 ; for a meta-analysis regarding bilingual immigrant children see Prevoo, Malda, Mesman, & van IJzendoorn, 2015). Early vocabulary is thus relevant when assessing developmental trajectories and risks (Henrichs et al., 2011 ; Lee, 2011 ; Ullrich & von Suchodoletz, 2011). Frequently discussed environmental characteristics influencing early vocabulary include type and quality of care (e.g., Ebert et al., 2013 ; Rodriguez & Tamis-LeMonda, 2011), interaction patterns of caregivers that might differ according to the child’s gender (Johnson, Caskey, Rand, Tucker, & Vohr, 2014 ; Lovas, 2011 ; Sung, Fausto-Sterling, Garcia Coll, & Seifer, 2013), and the mono- or multilingual composition of the language input children receive (e.g., Byers-Heinlein, 2013 ; Quiroz, Snow, & Zhao, 2010). In this study, we assessed the predictive value of gender, type and duration of early care, and monolingual versus bilingual family environment for the size and composition of 2-year-olds’ expressive German vocabulary. 

 Biological sexes and socially constructed genders have been discussed with regard to both presumed differences in language acquisition capacity or speed (Berglund, Eriksson, & Westerlund, 2005 ; Bornstein, Hahn, & Haynes, 2004 ; Hollier et al., 2013 ; Leaper & Smith, 2004) and systematically differing interaction patterns of adult caregivers’ speech directed at (baby) boys and girls (Johnson et al., 2014 ; Lovas, 2011 ; Sung et al., 2013). Contrary to popular perception, the child’s gender usually only explains about 1% to 3% of reported variance in vocabulary size or related variables (Ardila, Rosselli, Matute, & Inozemtseva, 2011 ; Szagun, Steinbrink, Franik, & Stumper, 2006 ; for a review see Hyde, 2014). This makes gender differences likely to be detectable in large samples only (e.g., Berglund et al., 2005 ; Bornstein et al., 2004 ; Leaper & Smith, 2004), but even a recent study that included more than 5,000 one- to six-year olds did not find reliable differences with regard to boys’ and girls’ language skills (Luijk et al., 2015). Thus, the existence and stability of gender differences in language acquisition patterns and/or speed, especially at an early age, is questionable. 

 Additionally, the direction of the found differences is often ambiguous, proclaiming advantages for boys or girls with regard to different language-related abilities and at different ages (e.g., Bockmann & Kiese-Himmel, 2006 ; Leaper & Smith, 2004). Still, presumed and measured gender differences frequently result in separate statistical norms for boys and girls (e.g., Bockmann & Kiese-Himmel, 2006 ; Fenson et al., 2008). The selective relevance of children’s gender in interaction with socio-economic characteristics, such as maternal education and parental stress levels, has only recently gained researchers’ attention (e.g., Barbu et al., 2015; Harewood, Vallotton, & Brophy-Herb, 2016 ; Vallotton et al., 2012 ; Zambrana, Ystrom, & Pons, 2012). Possible interactions of gender and other factors, such as characteristics of the care environment, are highly relevant and underresearched. This study assesses potential gender differences in vocabulary size or composition in an educationally homogeneous population at 2 years of age and further investigates whether such differences might be qualified by interactions with other environmental factors. 

 Studies investigating the effects of type, onset, duration, and quality of early childcare often have to deal with confounds of care quality and children’s individual and family characteristics (e.g., Belsky, Bell, Bradley, Stallard, & Stewart-Brown, 2007 ; Belsky & Pluess, 2012 ; National Institute of Child Health and Human Development, 2006; NICHD Early Child Care Research Network, 2001; Sylva, Stein, Leach, Barnes, & Malmberg, 2011). Within the variety of socio-economic status (SES)—related variables, parental and specifically maternal education has been shown to have strong influence on the language input provided and thus on children’s vocabulary acquisition (e.g., Hoff, 2013 , Magnuson, Sexton, Davis-Kean, & Huston, 2009 , but for contradictory results see also Letts, Edwards, Sinka, Schaefer, & Gibbons, 2013 ; Luijk et al., 2015). Previous research has also demonstrated that the relative influence of family-related factors (e.g., parental education and parenting quality) is larger than the influence of daycare related variables (Belsky, Vandell et al., 2007; Ebert et al., 2013 ; NICHD, 2006; Pinto, Pessanha, & Aguiar, 2013). In the last decades research has concentrated on compensatory efforts, demonstrating substantial developmental gains, specifically for disadvantaged children in high-quality daycare arrangements (e.g., Magnuson, Ruhm, & Waldfogel, 2007 ; for reviews see Burger, 2010 ; Jalongo & Sobolak, 2011) or for high-quality child-caregiver interactions (Vernon-Feagans, Bratsch-Hines, & The Family Life Project Investigators, 2013), while emphasizing the cumulative negative effects of social disadvantages (Ebert et al., 2013). We thus know that the increase in school success reported for high-quality care environments is mediated at least in part by the high-quality language input provided specifically for children at risk due to social disadvantages (Burger, 2010 ; Fram, Kim, & Sinha, 2012 ; Magnuson et al., 2009 ; Murray, Fees, Crowe, Murphy, & Henriksen, 2006 ; Pinto et al., 2013). Less well-investigated is the question whether differences in early care arrangements can be associated with differences in vocabulary acquisition in the absence of educational family disadvantages. 

 This study examines expressive vocabulary in a group of German-speaking 2-year-old children, who are homogeneous with regard to high parental education as well as employment status. These population characteristics enable us to assess predictors of vocabulary acquisition in the absence of explicit social and educational family-related risks. Also, the children attending early daycare were recruited exclusively from state-regulated centers where the standards of early education are monitored by governmental institutions to ensure high-quality care. While our study did not directly assess quality of interaction in daycare or family settings, the structural quality of the included daycare facilities as well as the families’ educational backgrounds were very high and indicate overall advantaged upbringing conditions. Characteristics of daycare environments differ across cultures and countries. Therefore, research in a German setting expands current knowledge obtained in studies conducted predominantly in Sweden, the United States, and Great Britain (e.g., Broberg, Wessels, Lamb, & Hwang, 1997 ; NICHD, 2006; Sylva et al., 2011). In this way, our study contributes to the discussion on the influence of early center-based daycare on early German expressive vocabulary acquisition in the absence of pronounced educational disadvantages. 

 Children’s vocabulary comprehension and production develop in exchange with the people a child interacts with. The early lexicon is thus shaped by the culture and environment that surround a child (Tardif et al., 2008). If children are regularly exposed to more than one language, their lexical abilities will develop according to the input received in each one of them (e.g., Bohmann et al., 2009; De Houwer, Bornstein, & Putnick, 2014 ; Hoff et al., 2012 ; Place & Hoff, 2011 ; Rinker, Budde-Spengler, & Sachse, 2016 ; Song, Tamis-LeMonda, Yoshikawa, Kahana-Kalman, & Wu, 2011 ; for a review see Gatt & O’Toole, 2016 ; Hammer et al., 2014). A small to medium vocabulary disadvantage for bilingual children has been reported when only one language is considered and has been linked to reduction of input when the total language input is divided between two languages (Bialystok, Luk, Peets, & Yang, 2010 ; Cote & Bornstein, 2014 ; Hoff et al., 2012 ; Junker & Stockman, 2002 ; Klassert, Gagarina, & Kauschke, 2014 ; Quiroz et al., 2010 ; Thordardottir, 2011 ; for a review see Unsworth, 2013). Multilingual or foreign language family environments in Germany are very often confounded with specific characteristics of the social environment, including higher incidence of poverty, educational disadvantages, and discrimination (e.g., Kigel, McElvany, & Becker, 2015). One recent study evaluated the early productive vocabulary in bilingual Turkish-German children aged 24 to 36 months finding much lower number of German versus Turkish items (Rinker et al., 2016) but comparable total numbers when both languages were considered. However, the Turkish speaking parents involved displayed relatively low SES and disadvantaged educational backgrounds typical for families of Turkish descent, especially in larger German cities. Therefore, which differences between mono- and bilingual children’s vocabulary actually do exist in the absense of educational disadvantages is an underresearched question with regard to German speaking children. In this study, we were able to evaluate early German expressive vocabulary in a small subgroup of bilingual children who were comparable to the monolingual group with respect to the educational background and employment status of their parents. 

 We investigated early lexical acquisition via parental report using a vocabulary checklist. The instrument employed in this study, Parents’ Responses (Eltern Antworten, ELAN; Bockmann & Kiese-Himmel, 2006), is a commonly used screening tool in Germany (Ullrich & von Suchodoletz, 2011). Thus, appropriate normative data for a standardization popualtion exist. ELAN, just as the internationally better known MacArthur-Bates Communicative Development Inventories (CDI; Fenson et al., 2008), assesses children’s productive vocabulary by asking parents (or sometimes teachers) to indicate which words of a preselected list a child speaks at a given point in time. Parental reports are directly related to language skills measured by other means, such as laboratory assessment, and are considered very reliable when identifying children at risk for language delays (Rowe, Raudenbush, & Goldin-Meadow, 2012 ; Ullrich & von Suchodoletz, 2011). Also, prior analyses of an extension of the current dataset indicated that ratings from two parents and from a parent and a teacher both reach high inter-rater reliability and agreement (Stolarova, Wolf, Rinker, & Brielmann, 2014 ). 

The evidence briefly reviewed above shows that early expressive vocabulary is influenced by the interaction of a variety of factors. In this study, children’s productive vocabulary at 24 months is assessed in an educationally homogeneous German-speaking group via parental report. The comprehensive statistical analysis based on mixed-effects regression models takes random effects of child and word into consideration to control for variance in the data caused by unsystematic inter-individual and inter-word differences. In this way, the model reveals general influences of theoretically grounded predictors (“fixed effects”) on the overall probability to speak any of the 250 ELAN-words. Below, the following predictors and their interactions are considered: gender of the child, type of care, and mono- versus bilingual family environment. In addition, duration of care in months and its relation to vocabulary size were investigated.

## Method

### Research Instruments and Procedure

 Participating children and parents (*N* = 58) were recruited from two middle size German cities and their surroundings. Parents responded to open advertisements at childcare centers (*n* = 8) and local media. Data collection took place within a period of two days before or after a child’s second birthday (*M*_age_ = 730.20 days, *SD* = 2.01). The number of spoken words was assessed on the basis of ELAN, the German lexical checklist for parents (Bockmann & Kiese-Himmel, 2006). ELAN consists of 250 words in 17 semantic categories, derived and pre-selected from the empirically determined expressive vocabulary of German speaking children (see Appendix 1 for an excerpt from ELAN). For each word, parents needed to check whether a child actively produces a certain word (*ja*, German for *yes*), or does not (*nein*, German for *no*). If the parents do not make a clear indication by checking one of the boxes, the answer is counted as missing. In addition, parents provide examples of their child’s utterances in a few open questions at the end and answer basic demographic questions at the beginning of the questionnaire. Study-specific parent and teacher questionnaires were also employed to collect further information on the educational and language backgrounds of the parents and teachers involved. For the purpose of the present analysis, vocabulary data provided by the parents who also answered the demographic questions (40 mothers, nine fathers, and two pairs of both parents) are considered. 

### Study Population

Vocabulary ratings were initially obtained for 58 2-year-old children (32 girls, *M*_age_ = 730.20 days, *SD* = 2.01, 24 months ± 2 days). Seven data sets were excluded from analyses to guarantee high data quality and a homogenous health status of the sample. Four data sets were excluded to ensure that all data stems from a group of normative developing children without any indication for language delays or health risks (three children with substantial risk for specific language delays, i.e., with scores below the 10th percentile of the standardization population, one bilingual; one child in daycare). Data of one girl in daycare was excluded due to her premature birth prior to the 26th week of gestation. Two data sets were excluded due to more than five missing answers (less than 2% of items) on the vocabulary checklist. Lastly, one child was excluded because he had started daycare only 2 months prior to testing and could not be assigned to either of the two care comparison groups (see below). Thus, data provided by parents of 51 children (27 girls) were included in the analyses.

At the time of testing, 32 children had experienced regular non-parental, center-based care for at least 6 months. We will refer to these children as the daycare group. Weekly daycare varied between the categories 11 to 20 hours (*n* = 5) and more than 20 hours (*n* = 27). All children attended daycare within a 5-days-a-week program. The duration of daycare experienced prior to testing at the age of 2 years varied between 6 and 22 months.

Children who were cared for exclusively by their parents (*n* = 19) and had no formal daycare experience will be referred to as the parental-care group. Children were also included in the parental-care group if they experienced some form of irregular and informal non-parental care (e.g., playgroups or babysitters) up to a maximum of 12 hours and up to three times per week. A summary of the demographic characteristics for the study population as well as for the two care subgroups is provided in Table 1.

**Table 1.** Population Characteristics

	Total	Daycare	Parental-care
	*N* (%)	*n* (%)	*n* (%)
Total	51	32	19
Data provider mother	40 (76.9)	25 (78.1)	15 (78.9)
Female	27 (52.9)	20 (62.5)	7 (36.8)
Firstborn^a^	36 (70.6)	21 (65.6)	15 (78.9)
Bilingual	12 (23.5)	9 (28.1)	3 (15.8)
Two-parent household	44 (86.3)	25 (78.1)	19 (100)
Highest sec. education^b^: mothers	42 (82.4)	26 (81.3)	16 (84.2)
Highest sec. education^b^: fathers	38 (74.5)	24 (66.7)	14 (73.7)
Mother employed	30 (58.8)	26 (81.3)	4 (21.1)
Father employed	50 (98.0)	32 (100)	18 (94.7)

*Note*. Percentages in brackets are group-based (column-wise). sec.: secondary. ^a^Including two pairs of firstborn twins, all four children were counted as firstborns. ^b^Refers to German university entrance certificate (Abitur) or a foreign equivalent, see footnote 1 for further explanations); all parents received further professional training and/or completed a higher education degree.

Taking the specifics of the German educational system into account, parental education levels were compared considering the highest secondary education degree obtained. The category reported by the vast majority of the parents was the German university entrance certificate (Abitur) or a foreign equivalent (see Table 1)^1^. In addition, all parents had received further professional training and/or completed a higher education degree. At the time of testing, mothers were either employed (*n* = 33), on parental leave (*n* = 17), or pursued a university degree (*n* = 2). All but one father were employed, the father who reported unemployment had only recently moved to Germany. No parent reported current involuntary unemployment. Income distribution was not assessed directly in this study. Taken together, the demographics indicate a non-representative, advantaged educational background and employment status of the participating families. While we did not collect specific income information from the parents, about the income situation of the families we can infer: Our sample did not include involuntarily unemployed parents, and children below the age of 3 years were only admitted into state-regulated daycare centers at the time and place of data collection if their parents were working or studying and children cared for at home had a family income allowing one parent to stay on parental leave for at least two full years after the child’s birth.

All children actively spoke German and listened to it on a daily basis. For 39 of them the family environment was monolingual German (subsequently referred to as monolingual children). In contrast, 12 children spoke another language with at least one parent (nine belonging to the daycare group, three to the parental-care group). One of those children (a girl attending a whole day daycare program for more than 11 months prior to the assessment) was raised in a trilingual family environment; her parents spoke two different languages other than German with their daughter, but communicated in German with each other. We included this girl in the group of 11 other bilingual children, as she was actively producing words only in German and her mother’s native language and was not yet speaking her father’s native language. The small subgroup of bilingual children constitutes a convenience sample recruited along with the monolingual group.

Testing was conducted exclusively in German. All multilingual parents demonstrated excellent understanding, speaking, and reading/writing skills during testing. Due to the lack of standardized questionnaires, we were not able to collect vocabulary information for all languages spoken by our multilingual participants but analyzed their children’s German expressive vocabulary only. For a summary of the bilingual children’s language backgrounds and information regarding language contact distribution, as well as a detailed table on parental education in relation to multilingualism, see Appendix 1.

 At the time of testing, child care spaces for children under the age of 3 years were very limited in the region of testing and only accessible to working or studying parents. This is an additional factor explaining why families of lower educational and social backgrounds, for example, unemployed parents, are not represented in our sample (and are likely underrepresented in the younger age groups in daycare facilities in this region in general), specifically in the daycare sample. As shown in Table 1, this non-representative SES distribution also holds true for the parental-care group but for reasons not systematically assessed here. One main hypothesis is the overall higher willingness of higher educated and better-off parents to participate in voluntary research with children (for a general discussion see Bergstrom et al., 2009 ; Heinrich, Heine, & Norenzayan, 2010).

### Characteristics of the Participating Daycare Centers and Teachers

All participating daycare centers were state-regulated and funded. The group size in the daycare centers varied between nine and 20 children, the majority of children (70%) were cared for in a group with up to 10 children and at least two daycare teachers were present at all times. A total of 24 daycare teachers primarily responsible for the participating children participated in the study and provided information on their own professional training and experience, four of them evaluated more than one child. All of the participating teachers were female native speakers of German, and all of them reported regular as well as recent participation in continuing education courses, including state-regulated courses on early language acquisition. All but one daycare teacher had completed a vocational degree in early child-care, the other teacher held a degree in nursing. Even though interaction quality was not directly evaluated, teachers’ vocational and further trainings, group sizes, child-to-teacher ratios, and governmental funding associated with strict control of the facilities taken together indicate relatively high structural quality of non-parental care in our daycare group.

### Analysis

 The complete data set is openly available at https://osf.io/vi28r/, a table displaying all estimated probabilities for boys and girls as well as mono- and bilingual children for each of the ELAN words can be accessed as a spreadsheet at https://osf.io/j69vc/; the analysis code is provided at https://osf.io/6e58y/. The dependent variable of interest here was the score *spoken*: yes (1) or no (0) for each of the ELAN words. We used mixed-effects logistic regression models (Baayen, 2008 ; Baayen, Davidson, & Bates, 2008) to investigate the influence of child related and environmental factors on expressive vocabulary. In this approach, the log of the ratio (logit) of spoken to unspoken words is the response variable. It is predicted from fixed (e.g., group, gender, duration of daycare) and random effects (child, word). Logits are equivalent to proportions but meet the mathematical requirements of the linear model. Outcome probability is assumed to vary randomly according to random effects (here: word and child), while at the same time the fixed effects of one or more predictors are assessed. This approach is especially useful when considering small and heterogeneous subgroups and relatively large item lists, as is the case in this study, because it modestly enhances power and takes inter-individual random variability into account. 

 The theoretically relevant predictors considered in this analysis were: daycare or parental-care (Group), male or female child (Gender), and mono- or bilingual family environment (Bilingual). Continuous predictors were the education level of the father (Education of Father) and the duration of daycare children in the daycare group had experienced (Duration of Daycare in Months). Education of the mother is also a theoretically important predictor of early vocabulary. However, we were unable to include it in this analysis, since it did not vary to a sufficient degree in the present sample (see Table 1 and Appendix 1). Similarly, the constellation of siblings (birth order, number of siblings or number of older siblings) was not included, as no informative predictor that was sufficiently independent from other predictors could be derived for this sample. The lmer function of the R package lme4 (Maechler, Bolker, & Walker, 2014) was used to conduct the analyses. 

**Figure 1.**
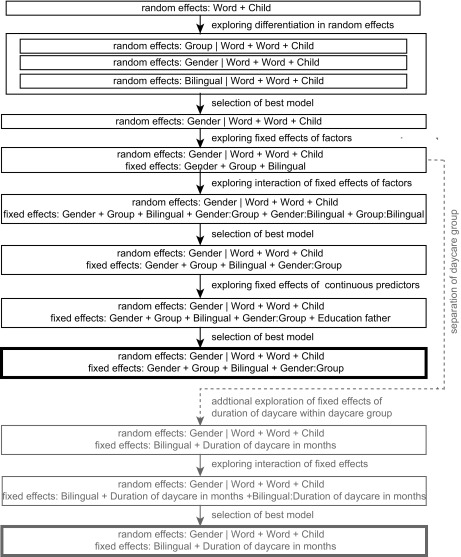
Flowchart displaying sequence of linear mixed models applied. Main analyses regarding the entire population are displayed in black, separate analyses for the daycare group are shown in gray. The best model was selected by removing non-significant predictors and likelihood ratio tests.

The best-fitting model was obtained sequentially: One cluster of predictors was added to the model at a time. Likelihood ratio tests ensured that the goodness of fit improved while taking costs of extra parameters into account. Figure 1 illustrates the sequence of models applied as follows: First, children (Child) and items (Word) were set as random factors for the initial model in order to account for random inter-individual and inter-word effects. Second, we explored whether the random effect of word varied according to the factorial predictors: Gender, Bilingual, and Group. Third, the factors Gender (reference level = female), Group (reference level = parental care), and Bilingual (reference level = false) were added to the best-fitting random effects model. Fourth, the continuous predictor Education of Father (reference level = lowest education) was added.

To test whether the expressive vocabulary of 2-year-old mono- and bilingual children experiencing regular daycare was predicted by the duration of daycare in months prior to data collection, we conducted a separate set of analyses including the predictor Duration of Daycare in Months (see Figure 1).

To summarize, random effects of child and word served to control for variance in the data caused by unsystematic inter-individual and inter-word differences. Exploration of estimated random intercepts for different words allowed identification of probabilities that a specific ELAN word is spoken. Fixed effects revealed the general influence of the predictors considered on the overall probability to speak any ELAN word.

 To illustrate the observed fixed effects, 95% confidence intervals (CIs) for proportions were calculated according to the groups of interest. The R package PropCIs (Scherer, 2014) was used to calculate these CIs. To relate results obtained for probabilities via mixed-effects models to the absolute number of words spoken and to the norms provided in the ELAN manual for 2-year-old boys and girls, we also calculated 95% CIs around the average number of words spoken in those subgroups of children meaningfully different according to the final mixed-effects model obtained earlier. 

## Results

### Expressive Vocabulary Predictors for the Entire Population

The final model’s estimated coefficients, their standard errors and z-values are displayed in Table 2. Collinearity was not observed between the predictors of this model, all correlations between predictors (ρ ≤. 25, and κ = 8.59) provided evidence that predictors varied independently from each other. The final model predicted the data better than the basic model which only included random effects, χ^2^ = 22.89, *p* < .001. In brief, children’s German expressive vocabulary size at the age of 2 years was predicted significantly by their bi- or monolingual language acquisition environments, and by the interplay between children’s gender and the type of early care they had experienced. This also means that children’s gender, the type of early care they had experienced prior to testing, or their fathers’ educational level did not independently improve predictions for productive vocabulary at the age of 2 years.

**Table 2.** Variance for Random Effects and Estimates, Standard Errors (*SE*s), and *z*-Values for Fixed Effects in the Final Model for the Entire Study Population

		Variance	Estimate	*SE*	*z*
Random effects	Word	3.17			
	Gender|Word	0.21			
	Child	1.94			
Fixed effects	(Intercept)		1.49	0.36	4.10***
	Gender		0.07	0.52	0.13
	Group		2.26	0.63	3.60**
	Bilingual		-1.77	0.47	-3.73***
	Group : gender		2.61	0.86	3.06***

*Note*. Reference levels for factors were: Gender = female, Group = daycare, Bilingual = false. **p < .01; ***p < .001.

### Random Effect Structure

The top row of Table 2 shows the random effects included in our final model. A considerable amount of variance in the probability that a particular word was rated as spoken can be attributed to differences between words, likely due to differences in difficulty and/or frequency of the words. Similarly, a high proportion of variance in the likelihood to speak any of the ELAN words was explained by inter-child variability, a likely and predictable illustration of the high inter-individual variability in early language acquisition. The systematic effects of the assumed and tested predictors reported below emerge and remain meaningful after statistically controlling for the random effects of word (item) and child.

**Figure 2.**
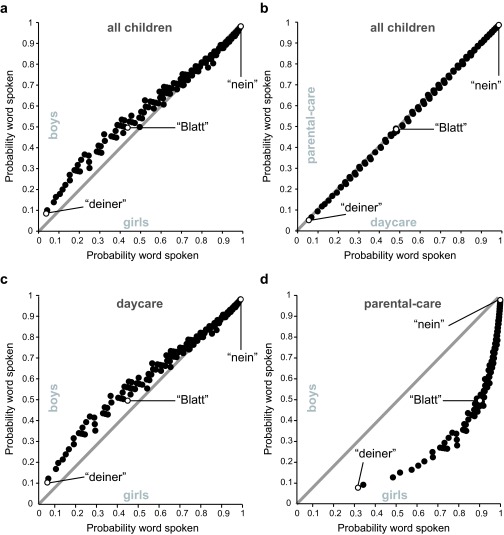
Probability that any word in the Eltern Antworten (EL AN) questionnaire is spoken based on estimates of random effects. Estimates in the top panels were derived from the model without fixed effects and random effects for Gender|Word (a), or Group|Word (b). Estimates in the bottom panels were derived from the final model and show random effects of Gender|Word separately for children in daycare (c) and in parental-care (d). The gray line marks equal probabilities for both subgroups in each panel. Data points of reference words re-appearing at similar places throughout are filled in white. The exemplarily displayed words translate to: *deiner* = *yours*, *Blatt* = *leaf*, *nein* = *no*. A list for all probabilities per word is available for further analyses at https://osf.io/j69vc/

Systematic differences between boys and girls were evident in a modulation of the random effect of words (as indicated by the significant term Gender|Word). That is, girls and boys differed in the probability to speak a certain word and thus in the presumed composition of their early vocabulary but not in the general number of spoken words (see below). Figure 2a illustrates this difference as well as the fact that most of the 250 ELAN words were spoken with similar probability by boys and girls, while there was large variance between words.

**Figure 3.**
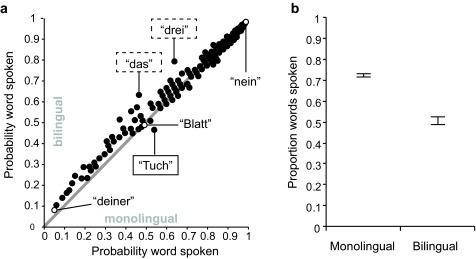
Probability that any word in the EL AN (Eltern Antworten) questionnaire is spoken based on estimates of the random effect of Bilingual|Word (a) and proportions of spoken words according to the fixed effect of bilingualism (b). Estimates of random effects were derived from the model without fixed effects. The gray line marks equal probabilities for both subgroups in each panel. Data points of reference words re-appearing at similar places throughout are filled in white. The exemplarily displayed words translate to: *deiner* = *yours*, *Tuch* = *cloth*, *Blatt* = *leaf*, *das* = *the*, *drei* = *three*, *nein* = *no*. A list for all probabilities per word is available for further analyses and is accessible at https://osf.io/j69vc/. Error bars in (b) denote 95% CI s for proportions

Bilingual and monolingual children differed with regard to the particular words they spoke (*var* = 271, comparison to initial model: χ^2^ = 11.86, *p* = .003). Figure 3a shows differences and commonalities in the probabilities that individual ELAN words were spoken by mono- and bilingual children.

The fit of the model that allows the random effect for word to differ between mono- and bilingual children was not better compared to the one including gender, χ^2^ = 0.0, *p* = 1. Hence, we selected the latter to continue analyses, since the gender of a child represents a more basic characteristic, and also because our sample included only a limited number of bilingual children (12) but a similar and higher number of boys and girls (27 girls and 24 boys).

Whether a child was cared for at home (parental-care group) or had regular daycare experience (daycare group) did not have a modulating effect on which words children were most and least likely to speak (see Figure 2b), χ^2^ = 0.17, *p* = .92.

### Fixed Effects

**Figure 4.**
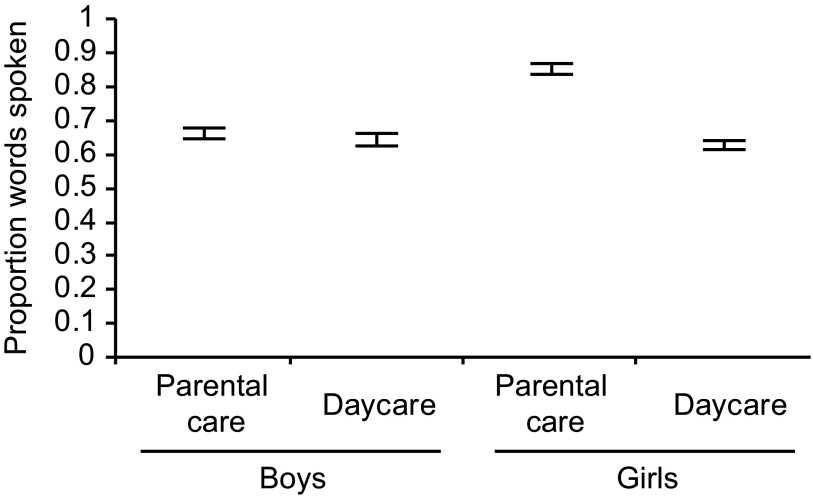
Proportions of spoken words according to the interaction of Gender and Group. Error bars denote 95% CI s for proportions.

In contrast to the random effects, for example, of word-that is, probabilities for individual words to be rated as actively spoken, fixed effects identify predictors for the probability that any ELAN word is spoken. Thus, fixed effects refer more directly to the quantity of spoken words also known as vocabulary size. The (Intercept) estimate refers to children’s average probability to speak a word at a reference level, here: girls, daycare group, monolingual, lowest education of the father. This probability decreased for bilingual children (see Figure 3b). The influences of gender and group interacted: Boys in daycare and boys in exclusively parental care did not differ from the reference group of girls in daycare, but girls in the parental-care group had a somewhat larger vocabulary size than all other children (see Figure 4).

### Effects of Daycare Duration

To examine the potential influence of the duration of daycare experience prior to testing on children’s vocabulary, we separated the data of the children in daycare (*n* = 32) after determination of random effects (see Figure 1). As the smaller number of children does not allow taking all available predictors into consideration without basing analyses on data of individual children, we only entered two predictors of interest: Bilingualism and Duration of Daycare in Months in the initial models. Again, collinearity was not observed, as the correlation between predictors was low, ρ = -.19. The final model’s estimated coefficients, their standard errors, and *z*-values are displayed in Table 3.

**Table 3.** Variance for Random Effects and Estimates, Standard Errors (*SE*s), and *z*-Values for Fixed Effects in the Final Model for the Daycare Group

		Variance	Estimate	*SE*	*z*
Random effects	Word	3.31			
	Gender|Word	0.53			
	Child	1.50			
Fixed effects	(Intercept)		0.24	0.73	0.33
	Bilingual		-2.02	0.50	-4.05***
	Months in daycare		0.12	0.06	0.03*

*Note*. Reference levels for factors were: Gender = female, Bilingual = false. *p < .05; ***p < .001

**Figure 5.**
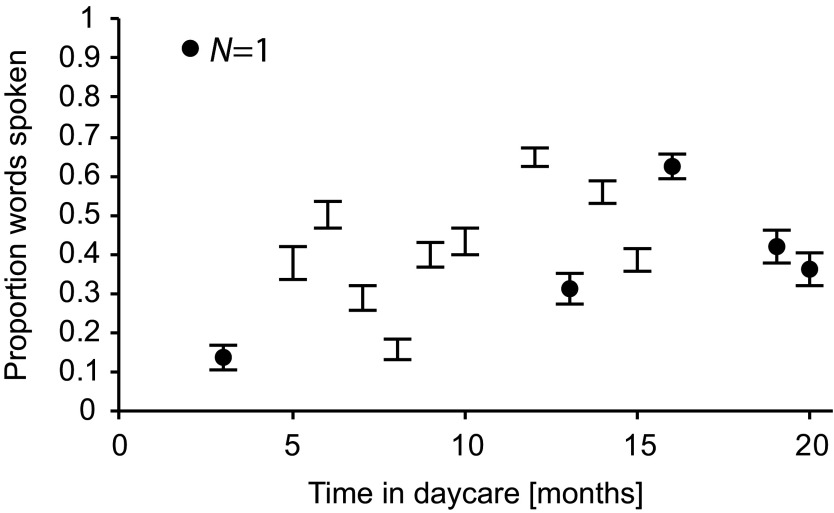
Proportions of spoken words according to duration of daycare in months for the children in the daycare group. Black dots mark CIs based on data of an individual child. Error bars denote 95% CI s for proportions.

**Figure 6.**
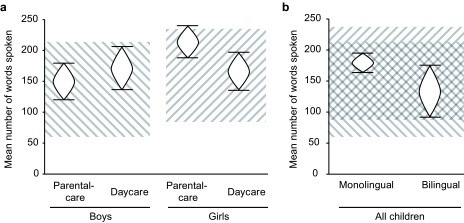
95% CI s around mean number of words spoken by boys and girls in different care groups (a) as well as mono- and bilingual children (b). Cross-hatched areas mark ±1 SD around the mean number of words spoken by 24-month-old boys (lines from top-left to bottom-right) and girls (lines from bottom-left to top-right) in the norm sample of the Eltern Antworten (EL AN) questionnaire’s manual (Bockmann & Kiese-Himmel, 2006).

The model fit improved by adding the predictors Bilingual and Duration of Daycare in Months, χ^2^ = 243.58, *p* < .001, but not by including the interaction between both, χ^2^ = 0.03, *p* = .86. Thus, bilingualism and duration of daycare independently predicted expressive German vocabulary in the daycare group. The reference group-that is, the values from which the model calculates changes, consisted here of monolingual children with (fictive) minimal daycare duration of 0 months. With increasing time spent in daycare, the probability to speak any word increased (see Figure 5), such that, for example, a child having spent 12 months in daycare (the median and mean value in this sample) would have had a 12% increase in productive vocabulary compared to a child having spent 6 months in daycare. Bilingualism again negatively predicted expressive German vocabulary size, such that a bilingual child experiencing regular non-parental daycare would have had a decreased average probability to speak any of the German ELAN words in comparison to a monolingual child with the same daycare experience. As shown in Figure 6 and explained below, vocabulary size of both, bilingual and monolingual children varied within the expected normative range.

### Average Number of Words Spoken and Relation to ELAN Norms

 The final mixed-effects model obtained in our analyses showed that there are meaningful differences regarding children’s probability to speak any ELAN word, an estimate of vocabulary size. Figure 6 illustrates how these effects correspond to differences regarding the absolute number of words reported to be spoken: Girls in parental care speak on average more words than all boys, and girls in daycare and bilingual children speak on average less words than monolinguals. Comparison with means and standard deviations (SDs) provided in the ELAN Manual (Bockmann & Kiese-Himmel, 2006) for the standardization population of 24-month-old monolingual German boys and girls shows that the mean number of words spoken in all subgroups in this study falls within ± 1 SD of the norm. This illustrates that all children in this study exhibited at least normative average vocabulary size. It also shows that the girls in parental care, for whom a difference in vocabulary size compared to the three other groups was detected, had the largest vocabulary: The 95% CI surrounding the means of this group extended slightly above 1 SD of the standardization population (see Figure 6a). 

## Discussion

The main purpose of this study was to assess a series of potential predictors for expressive vocabulary development in a group of 2-year-old German-speaking children in two different early care settings: exclusive parental care and center-based daycare. In this way, we examined whether either of these care environments is associated with specific early vocabulary advantages or disadvantages. We also assessed whether boys and girls, as well as mono- and bilingually raised German-speaking children, differ systematically with regard to expressive vocabulary size or composition. The children participating in this study came from educationally homogeneous, advantaged family backgrounds. This allowed us an assessment of early vocabulary in the absence of pronounced disadvantages and also diminished possible confounding effects of family background and quality of early care. In addition, we restricted the age range to ± 2 days around the children’s second birthday and were thus able to assess expressive vocabulary in a group highly homogeneous, not only with regard to educational background of the parents but also to age. The use of logistic mixed-effect models allowed us to analyze potential predictors of vocabulary size while controlling for differences between individual children and words. At the same time, systematic variation in random effects revealed meaningful divergences in the composition of vocabulary between subgroups of children. Finally, we related the fixed effects in our mixed-effects model to the duration of daycare and the absolute amounts and means of words spoken, and compared the vocabulary size in our study to the normative range reported in the manual of the employed assessment tool.

 Two-year-old girls and boys differed with regard to the probability to speak certain words and thus with regard to vocabulary composition (see Figure 2a for some examples) but exhibited very similar vocabulary sizes (see Figures 4 and Figure 6). Within our group of children with homogeneously high SES, the type of early care experience was not a meaningful predictor of vocabulary size or composition (see Figure 2b and Figure 4), but this main effect was modulated by an interaction (as discussed below). Neither exclusive parental care nor early center-based daycare settings were associated with specific disadvantages regarding children’s expressive vocabulary at 24 months. Rather, we found an overall average vocabulary size across care groups, genders, and for mono- and bilingually raised children. The educational level of the father did not contribute to the prediction of expressive vocabulary in our sample, with relatively high average paternal education, low variability of this potential predictor, and virtually no variability of maternal education (see Table 1). Given that we assessed children from homogeneous family backgrounds, the absence of differences with regard to vocabulary size and composition between the groups of children with different care arrangements before the age of 2 years is in accordance with previous research which has demonstrated that the influence of family characteristics and specifically of maternal education on language is stronger than the influence of care type (Belsky, Bell et al., 2007; Magnuson et al., 2009 ; NICHD, 2006; Pinto et al., 2013 ; Sylva et al., 2011). Future research could replicate and extend our finding by including larger and demographically more variable groups of children and by using a vocabulary assessment instrument that includes more words. For Germans this could be Fragebogen zur Erfassung der frühkindlichen Sprachentwicklung 

 (FRAKIS)—that is, questionnaire for the measurement of early childhood language development (Szagun, 2004), which measures productive vocabulary, sentence complexity, and length of utterance. Another German language parent report assessment tool for expressive vocabulary, syntax, and morphological skills is Elternfragebögen für die Früherkennung von Risikokindern (ELFRA-2)—parents questionnaires for the early recognition of children at risk (Grimm & Doil, 2000). 

 The gender of the 2-year-old children alone did not predict differences in vocabulary size. Considering the relatively small group of 2-year-old children examined here, the possibility that effects of gender on vocabulary size or other linguistic abilities might emerge at a later age or can be detected in larger samples cannot be excluded on the basis of our results. Our results are, however, in line with previous findings: If there is a (direct or indirect) gender influence on early expressive vocabulary at all, it is small. They are also consistent with recent findings reporting gender differences in language acquisition in low but not in high SES children (Barbu et al., 2015). The expected performance overlap between genders is large, making the relevance of such presumed differences for everyday communication and early childhood education at least questionable. 

 In our study, an interesting interaction between gender and type of care emerged. It showed that girls cared for at home and not attending daycare before the age of 2 years exhibited somewhat larger vocabulary size in comparison to all other children. Yet, all subgroups of children showed an average vocabulary size (see Figure 6). Due to limitations regarding the size of the subgroups (only seven girls did not attend daycare), this interaction has to be interpreted with caution. Also, we cannot make any conclusive claims about the underlying reasons for these differences, but they could relate to parental communication behavior (Bohman et al., 2009 ; Harris et al., 2010 ; Hart & Risley, 2003 ; Hoff, 2006 ; Rohacek et al., 2010) and complement recent reports on differential effects of environmental variables for boys and girls (Barbu et al., 2015 ; Berglund et al., 2005 ; Vallotton et al., 2012 ; Zambrana et al., 2012). 

 Judging by structural quality characteristics, such as teacher’s education background, group sizes and teacher-to-child ratios, daycare provided for our sample was likely of high quality. Researchers have argued that high-quality center based daycare is particularly beneficial for the development of socially and educationally disadvantaged children (Burger, 2010 ; Phillips & Morse, 2011), a group that was not assessed in this study. Nonetheless, we investigated whether vocabulary scores change according to the time children had spent in center based daycare before their second birthday (see Figure 4), since some studies have reported particularly beneficial effects of high-quality extensive daycare before children’s first birthday on children’s vocabulary up to the age of 5 years (e.g., Belsky, Vandell et al., 2007). Within children attending regular state-regulated daycare, we found increasing vocabulary size with increasing duration of prior daycare experience. The nature of this relation is correlational, it relies on cross-sectional data, and the assignment to very early versus later age at daycare entry is likely not random. Thus, we cannot argue that the prolonged daycare experience directly benefitted children’s expressive vocabulary at the age of 2 years. In light of previous research, however, we assume that the combination of a structurally high-quality daycare environment and the possibility for regular interactions with peers as well as with trained adult caregivers (Belsky, Bell et al., 2007; NICHD, 2006) have a positive impact on children’s early expressive vocabulary. Further investigations with larger and more diverse samples in longitudinal designs are needed to clarify whether and how high-quality early daycare might generally benefit vocabulary acquisition in young children in the absence or presence of social disadvantages. Young children with multilingual and/or non-German family language environments are of particular interest in this regard. 

Independent of care group, we found evidence for somewhat higher German expressive vocabulary size in monolingual compared to bilingual children. In addition, we found differences with regard to the composition of the early German vocabulary exhibited by mono- and bilingually raised 2-year-olds (see Figure 3a and Appendix 1for details). The bilingual children exhibited age-appropriate German expressive vocabulary (see Figure 6), and the differences between mono- and bilingual children were of medium size. We attribute these relatively minor differences in German expressive vocabulary between bilingual and monolingual children to overall high parental education, the absence of systematic differences in family background, mostly family environments with one German-speaking parent (10 out of 12), and the fact that nine out of 12 bilingual children experienced regular monolingual German high-quality daycare. However, there was somewhat larger variance in parental education for bilingual compared to monolingual families in our sample. Thus, we cannot conclude to what extent the differences in average German vocabulary size of mono- and bilingual children might be attributable to the small differences in parental education or to the bilingual language acquisition itself. But we provide evidence that at the age of 2 years, the differences between these mono- and bilingual children in vocabulary size and composition are small and thus unlikely to have negative long-lasting effects on everyday communication and language acquisition. Future research should assess the effects of these moderate early differences longitudinally to determine whether they tend to decrease as bilingual children spend more time in monolingual educational settings.

In conclusion, we found no differences with regard to the measured predictors of early vocabulary size or composition between groups of German-speaking children attending and not attending center-based daycare before the age of two years. No general gender differences regarding expressive vocabulary size for these children from a homogeneous, well-educated family background were found either. Girls in exclusively parental care exhibited somewhat larger average vocabulary sizes, compared to all other subgroups of children, but overall, all subgroups’ vocabulary size was at least average compared to the standardization population. Thus, both types of care environments seem to provide adequate levels of language input needed for successful early vocabulary acquisition under the investigated circumstances and specifically in the absence of social or educational family disadvantages. We also showed that bilingual 2-year-old children exhibit slightly lower expressive vocabulary when only one language, in this case German, is considered. In our study, this difference was unlikely to predict further educational disadvantages, since vocabulary size for all 12 bilingual children remained within 1 SD of the mean of the monolingual standardization population and thus cannot be considered different from it. This study expands current knowledge about relevant predictors of early vocabulary. It shows that in the absence of educational disadvantages prolonged high-quality early daycare experience is associated with larger vocabulary but also points to the fact that environmental characteristics, such as type of care, might affect boys’ and girls’ early vocabulary in different ways.

## Author Note

Funding for this study was provided by the Zukunftskolleg of the University of Konstanz. We thank the families who participated in this study and extend our gratitude to the researchers who assisted with data collection: Anna Artinyan, Katharina Haag and Stephanie Hoss. All parents, the heads of the daycare centers, and all daycare teachers involved in this study gave written informed consent according to the principles of the Declaration of Helsinki (see http://www.wma.net/en/20activities/10ethics/10helsinki/) prior to participation. Special care was taken to ensure that participants understood that their participation was voluntary and could be ended at any time without causing disadvantages to them, their children or the daycare centers.

## APPENDIX A

**Table A1.** Language Background of Bilingual Children

Maternal native language	Paternal native language	Gender	Group
German	Spanish (12 h/week)	female	daycare
German	Arabic (30 h/week)	female	daycare
German	Arabic (30 h/week)	female	daycare
Russian (11 h/week)	German	female	daycare
Russian (30 h/week)	German	male	daycare
German	Albanian (23 h/week)	female	daycare
German	Hebrew (26 h/week)	male	daycare
German	Italian (14 h/week)	female	daycare
Spanish (12 h/week)	German	female	daycare
German	English (45 h/week)	male	parental-care
Croatian (60 hours/week)	Croatian (60 h/week)	female	parental-care
German	Italian (35 h/week)	female	parental-care

*Note*. Numbers in parentheses give approximate weekly hours each child was exposed to another language than German as reported by the parents.

**Table A2.** Level of Parental Education According to Care Group for Mono- and Bilingual Children

		Daycare		Parental care	
Parent	Education level	*N* monolingual	*N* bilingual	*N* monolingual	*N* bilingual
Father	*Hauptschule*^a^	2	2	0	1
	*Realschule*^b^	3	1	2	2
	*Abitur*^c^ *or more*	18	6	14	0
Mother	*Hauptschule*^a^	1	0	0	0
	*Realschule*^b^	3	2	1	2
	*Abitur*^c^ *or more*	19	7	15	1

*Note*. ^a^corresponds to 9 years of schooling; ^b^corresponds to 10 years of schooling; ^c^corresponds to 12 or 13 years of schooling

Excerpt of the original German vocabulary questionnaire (ELAN)

After two pages of general questions about the child’s characteristics, the ELAN provides a checklist of word (see below for an excerpt). Here an unofficial translation of the instruction at the top of this list here: “In the following you will find a list of words. Please check all of the words that your child speaks. Correct pronunciation does not matter. Please check either the yes- or no-box for each of the words.” The list of 250 words is subdivided into 17 categories: body parts, vehicles/accessories, outdoors (shown above), toys/playground/symbolic figure, animals/parts of animals, clothes/requisites, furniture/household, activities, people, hygiene, qualities, pronouns/articles, auxiliary verbs, miscellaneous, quantities, question words. At the end of the ELAN, parents or teachers are asked for any other words the child speaks, for combination words it forms and to give three typical examples for the child’s utterances.
